# 

**Published:** 1906-06

**Authors:** 


Sixty-first Year.

VOL. LXI. JUNE, 1906. No. 11

### BUFFALO MEDICAL JOURNAL

Established 1845 by AUSTIN FLINT, M. D.

[illegible] OFFICE
MAY 28 1906

[illegible]

EDITOR:

WILLIAM WARREN POTTER, M. D.

ASSISTANT EDITORS:
WILLIAM C. KRAUSS, M. D. NELSON W. WILSON, M. D.

ASSOCIATE EDITORS:
JAMES WRIGHT PUTNAM, M. D. ERNEST WENDE, M. D.
JOHN PARMENTER, M. D. JOHN A. MILLER, PH. D.
HARVEY R. GAYLORD, M. D. MAUD J. FRYE, M. D.
JOHN A. RAFTER, M. D.

